# Integrated and Functional Genomics Analysis Validates the Relevance of the Nuclear Variant ErbB3_80kDa_ in Prostate Cancer Progression

**DOI:** 10.1371/journal.pone.0155950

**Published:** 2016-05-18

**Authors:** Mahmoud El Maassarani, Alice Barbarin, Gaëlle Fromont, Ouafae Kaissi, Margot Lebbe, Brigitte Vannier, Ahmed Moussa, Paule Séité

**Affiliations:** 1 Equipe 2RCT, Université de Poitiers, Faculté des Sciences Fondamentales, Pôle Biologie- Santé, 1 rue G. Bonnet, 86073, Poitiers cedex 9, France; 2 Centre Hospitalier Universitaire Bretonneau, Laboratoire d’Anatomopathologie, INSERM U1069, 37000 Tours, France; 3 LTI Laboratory, Abdelmalek Essaadi University, ENSAT, BP 1818, 90 000 Tangier, Morocco; Innsbruck Medical University, AUSTRIA

## Abstract

The EGF-family of tyrosine-kinase receptors activates cytoplasmic pathways involved in cell proliferation, migration and differentiation in response to specific extracellular ligands. Beside these canonical pathways, the nuclear localization of the ErbB receptors in primary tumours and cancer cell lines led to investigate their role as transcriptional regulators of cancer genes. The nuclear localization of ErbB3 has been reported in various cancer tissues and cell lines but the nuclear functions and the putative correlation with tumour progression and resistance to therapy remain unclear. We first assessed ErbB3 expression in normal and tumour prostate tissues. The nuclear staining was mainly due to an isoform matching the C-terminus domain of the full length ErbB3_185kDa_ receptor. Nuclear staining was also restricted to cancer cells and was increased in advanced castration-resistant prostate cancer when compared to localized tumours, suggesting it could be involved in the progression of prostate cancer up to the terminal castration-resistant stage. ChIP-on-chip experiments were performed on immortalized and tumour cell lines selected upon characterization of endogenous nuclear expression of an ErbB3_80kDa_ isoform. Among the 1840 target promoters identified, 26 were selected before ErbB3_80kDa_-dependent gene expression was evaluated by real-time quantitative RT-PCR, providing evidence that ErbB3_80kDa_ exerted transcriptional control on those genes. Some targets are already known to be involved in prostate cancer progression even though no link was previously established with ErbB3 membrane and/or nuclear signalling. Many others, not yet associated with prostate cancer, could provide new therapeutic possibilities for patients expressing ErbB3_80kDa_. Detecting ErbB3_80kDa_ could thus constitute a useful marker of prognosis and response to therapy.

## Introduction

The EGF-receptors family consists of four tyrosine kinase (TK) receptors (EGFR/ErbB1, HER2/ErbB2, HER3/ErbB3 and HER4/ErbB4) with an extracellular ligand-binding domain, a transmembrane domain and an intracytoplasmic domain. Upon specific ligand binding at cell surface, ErbB receptors dimerize and trigger intracellular pathways involved in cell proliferation, migration and differentiation [1]. The deregulation of ErbB downstream pathways, through three mains mechanisms -increased receptor expression, increased ligand expression or activating mutation of the TK domain- is frequently involved in tumorigenesis [2]. In addition to their localization at the plasma membrane, ErbB receptors have also been described in the nucleus and various functions have been ascribed to the translocated ErbB receptors among which are cell proliferation, DNA repair and transcription control [3–8]. In this regard, the full-length ErbB1 receptor in the nucleus is associated with increased expression of proliferative and metastatic genes [9–11]. The expression of the pro-apoptotic and pro-angiogenic COX-2 protein is modulated upon ErbB2-binding on the *COX-2* promoter in mammary tumour cells [12]. ErbB2 also functions as a transcriptional co-activator of the Stat3 protein on the *CCND1* (Cyclin D1) promoter [13] and collaborates with ErbB1 to up-regulate *STAT1* gene expression [14]. The full length ErbB3_185kDa_ protein or short isoforms have been described in the nucleus of normal mammary epithelial cells [15] and Schwann cells [16] as well as in various human cancer cell lines: breast (SKBr3, MCF-7 et BT474), lung (H226 et SCC6) head and neck (SCC6 et SCC1) and colon (LoVo et CaCO2) [15, 17–19]. In H358 non-small cell lung cancer (NSCLC) and SKBR3 breast cell lines, ErbB3 has been shown to exert function as a co-transcriptional activator for *CCND1* gene expression [17,19]. The strong transactivation potential of ErbB3 relies almost exclusively on the C terminal domain of the receptor [19].

Prostate cancer (PCa) is the most common urologic malignancy and the second leading cause of cancer death in the industrialized countries. Tumour recurrence after surgical ablation or radiation therapy frequently occurs in a significant fraction of patients that develop disseminated aggressive disease. As PCa cells rely on androgen receptor (AR) activity for growth and proliferation, androgen withdrawal therapy has been endorsed in those advanced cases [20]. Beneficial effects are observed for about 24 months before the patient develops fatal castration-resistant-prostate-cancer (CRPC). Molecular mechanisms involved in therapy resistance still remain unclear. One explanation for a patient’s relapse after treatment could come from cross-talk between AR signalling and the epithelial growth factor family of receptors (EGFR) pathways [21]. The ErbB receptors and their ligands are expressed in the normal adult prostate to maintain the homeostasis of the organ and ErbB1, ErbB2 and ErbB3 are frequently increased during PCa progression [22]. In CRPC, increased ErbB2 receptor and/or ErbB1, ErbB2 or ErbB3-ligands expression is associated with poor prognosis and metastasis [23–25].

To by-pass the mechanisms of resistance to androgen-therapy, ErbB1 and ErbB2 TK-inhibitors have been tested alongside hormone-based therapy in patients with high grade, CRPC tumours. However, trials were not successful as tumour cells developed compensatory pathways by regulating ErbB3 expression and localization [18,26]. Indeed, several studies have reported the nuclear localization of ErbB3 in prostate tissues and PCa cell lines potentially correlated with disease progression [18,27].The nuclear ErbB3 protein could thus play a pivotal role in PCa progression and therapy resistance, but the molecular mechanisms involved still remain unexplored.

The current study demonstrates the functional relevance of ErbB3 nuclear localization in PCa and provides new clues for the understanding of pathways associated with cancer progression and therapy resistance.

## Materials and Methods

### Patients Tissue microarrays

Written informed consents were obtained from patients in accordance with the requirements of the institutional ethic committee of Centre Hospitalo-Universitaire de Poitiers that approved the study. 169 formalin-fixed, paraffin-embedded, archival tissues from patients treated at the “Centre Hospitalier Universitaire de Poitiers” for PCa, were included. 137 tissue samples were obtained from hormone-sensitive (HS) patients that underwent radical prostatectomy for clinically localised cancer, none of these patients received hormone deprivation therapy before surgery. Of these, 97 were stage pT2 and 40 pT3. The Gleason scoring was respectively 6 in 38 patients, 7 in 83 patients, and 8 or more in 16 patients. 32 tissue samples were obtained by trans-uretral resection from CRPC patients. Normal appearing prostate tissue from 50 patients treated by radical prostatectomy were also included. Immunostaining was performed using the following antibodies: ErbB3 C-ter (rabbit polyclonal sc-285, Santa Cruz Biotechnology 1/200), ErbB3 N-ter (Mouse monoclonal Ab-5 Clone H3.105.5, Neomarkers, 1/50), CCND1 (Rabbit monoclonal, clone EP12, DAKO, 1/50) and Ki67 (Mouse monoclonal, clone MIB-1, DAKO, 1/100). Negative controls were obtained by the use of an irrelevant antibody. Tissue from mantle zone lymphoma was used as positive control for Ki67 and CCND1. Slides were analysed by 2 observers in a blind fashion. For ErbB3C-ter, CCND1 and Ki67 staining, each tissue core was given a continuous value representing the percentage of tumour cells exhibiting nuclear staining. For each patient, the core with the highest percentage was selected.

### Cell lines

Human prostatic RWPE cells (ATCC^®^ CRL11609™), normal epithelial cells immortalized by HPV-18, were maintained in Keratinocyte Serum Free Medium supplemented with bovine pituitary extract (BPE, 0,05mg/ml) and human recombinant epidermal growth factor (EGF, 5ng/ml) (Invitrogen). Human prostatic LNCaP cell line (ATCC^®^ CRL1740™) derived from lymph node and PC3 (ATCC^®^ CRL1435™) cell line derived from bone metastasis, were grown in DMEM supplemented with 10% foetal calf serum and 1% penicillin/streptomycin.

### Western blotting

Protein extracts and western blot analysis were performed as described in [17]. Nuclear and non-nuclear extracts were prepared as follows: cells were resuspended in Tris–HCl 10 mM pH7.5, NaCl 120 mM and lysed with NP40 to a final concentration of 0.3%. After centrifugation for 5 min at 2500 g, supernatants were supplemented with EDTA 1 mM, DTT 1 mM, SDS 0.1%, PIC 1/50 and PMSF 1/100 to constitute the non-nuclear fraction. The pellet corresponding to nuclei was washed three times in Tris–HCl 10 mM pH 7.5, NaCl 120 mM, NP40 0.3% to eliminate cytoplasmic residues, then lysed as above.

### ChIP-on-chip

Cells were treated with 1% formaldehyde and chromatin extraction was performed with the Simple ChIP Enzymatic Chromatin IP kit (Cell Signaling Technology, CST) according to the manufacturer’s recommendation. The recovered chromatin was digested by Micrococcal nuclease to obtain chromatin fragments around 500pb size. At this step, a total DNA sample (Input) was collected for subsequent use. Immunoprecipitation was performed using anti ErbB3 C-ter antibodies (sc-285 or sc-7390, SCBT) or anti-H3 (positive control, Histone H3 antibody ChIP formulated #2650, CST) and for incubation with the non-relevant normal rabbit IgG (ChIP negative control). Total DNA and ChIP-DNA were amplified using the Whole Genome Amplification Kit (WGA-2, Sigma-Aldrich) and respectively labelled with Cy-3 or Cy-5 fluorochromes using BioPrime Array Genomic Labelling kit (Invitrogen). Labelled samples were hybridized on Human Promoters Microarrays (Agilent's Human Promoter ChIP-on-Chip Microarray Set 244K, P/N G4489A) according to the manufacturer’s recommendation.

### Normalization and analysis of microarray data

Data provided by Agilent high resolution DNA Microarray scanner were analysed using Feature Extraction 10.1 Software (Agilent Technologies). The fluorescence spots that failed to pass quality control were excluded. Signals of enrichment ratios (ChIP/Input) related to merged replicates were calculated using CoCAS software [28]. ErbB3-enriched promoters from three independent experiments were isolated using the peak detection algorithm in CoCAS followed by the enrichment scores calculated by measuring the effective peak area.

### Real-time quantitative PCR (qPCR) analysis

qPCR experiments were performed on ErbB3-immunoprecipitated or IgG-control DNA, using the GoTaq qPCR Master Mix (Promega) according to the manufacturer’s instructions on a 7500 Fast Real Time PCR system (Applied Biosystems). Relative enrichment was estimated after quantification of PCR signal by measuring the ratio IP ErbB3/IgG. List of ChIP-qPCR primers is given in Table 1.

**Table 1:** Primers sequences for ChIP-qPCR analysis.

Gene Promoter Name	Forward primers (5'-3')	Reverse primers (5'-3')
ACE	TAG-CTC-ATG-CCC-AAG-GAA-AC	GAT-GCT-GCC-ACT-GTC-ATT-TC
	GCT-AAG-CAA-CAT-GAG-CAG-GAT-C	AGA-CCC-TAC-ACA-ACT-GCA-TGG
AGR2	AAT-GCA-GGC-TGG-CTT-AAG-AC	AGG-ATG-TGC-AGG-TTT-GTT-CC
	TGG-AAC-AAA-CCT-GCA-CAT-CC	AAT-TAC-CTC-CTC-CTT-GCC-ATC-C
ARID1A	AAA-GCT-GAT-GGG-CCA-GTT-TC	AAG-AAC-AGA-CAG-CTC-CAG-ACG
	TGG-GTT-GGA-GGA-GAT-AAT-GTG-G	TGC-GAG-TTT-GAG-CAA-AAG-GG
CCND1	AAC-TTG-CAC-AGG-GGT-TG	ATT-TAG-GGG-GTG-AGG-TGG-AG
	AAA-GAA-GAT-GCA-GTC-GCT-GAG	TGC-AGT-AGG-GGA-CAA-CTA-GGA-A
	GGG-AAA-GAA-GAT-GCA-GTC-GCT-GAG-A	ATT-AAG-GGG-GTG-AGG-TGG-AG
CFTR	TTG-GCA-TTA-GGA-GCT-TGA-GC	GAG-ACA-ACG-CTG-GCC-TTT-TC
	TTT-AAC-CTG-GGC-AGT-GAA-GG	CGC-TCA-ACC-CTT-TTT-CTC-TG
CXCR3	AGA-TGG-GAG-GCT-CAA-AGG-TTG	AGT-GCT-CCA-AGA-GGC-ATT-TG
	AGC-TGA-AGT-CAC-AGG-GAG-AC	AAA-AAG-CAG-TGT-CCC-CAC-AG
ErbB2	TCC-CAG-ACT-TGT-TGG-AAT-GC	AAT-GGA-GGG-GAA-TCT-CAG-CTT-C
	AGA-CAT-CCT-GGG-CAA-ATT-GC	TCT-GGA-TGG-CCA-TCA-ATA-TCC-C
ErbB3	AGG-CAG-ATC-ATT-CCC-ATG-AC	CAC-CCA-CTT-TAT-CCT-CAG-CTT-C
	ACC-AAA-CAC-CCC-AAA-CTG-TC	TGT-GCC-TAA-GCC-CTT-TCT-TG
ERBIN	AGC-TGC-AAG-AGA-TAC-CTG-CTG	GCC-GAT-GGA-GTT-TCT-TTG-TAC-G
	TGA-AAA-AGC-TGC-CCT-GAG-AC	TCC-GAG-TCA-CAA-AGT-CCC-ATG
EBP1	AAT-AGC-CGC-CTG-ACA-ATG-TG	TCA-TCT-TCA-AGA-CGC-AGC-AG
	TCT-GAC-GGC-TGT-GAA-AGA-TG	CTG-CAA-TCC-GAC-ACC-AAA-C
FYN	TCC-TTT-ACT-ACC-ACA-GGC-ACA-G	TTG-TAG-ACT-GGA-CTG-TGG-TCT-G
	TGC-TTT-CTT-CAG-GCG-AAC-AG	CAC-AGT-GCA-CAA-GCT-TCT-AGA-G
GATA2	TCT-GGC-GTC-CGT-TTG-TCT-G	CAG-AGT-GGA-GTT-CCG-AGC-AG
	CGG-GAA-AGC-AGG-TAA-TTC-GC	TGC-GCT-GAA-ACT-GGG-AAT-AC
ID3	TGT-TGA-GGA-ATC-CGC-TCC-TTT-G	TGG-GGC-TGA-GTC-TTA-GAT-CAA-C
	TTG-CTT-GCC-TTC-TTG-TCA-CC	GGA-ATC-CCA-CTT-GGT-CAT-TTC-C
MAP3K14	AGT-CAC-TTG-TGC-AGC-CTC-AG	TTC-CAG-ACC-TGA-AAT-GGA-TGG-G
miR488	ATC-CTG-GAC-GGC-TAT-GAG-TAT-G	CAA-AGT-CCT-CCC-CTC-CGT-TC
	TGT-TGT-CAC-ACA-GCT-GTT-CC	CTG-CAA-TGA-AAA-GTC-GGG-GAT-G
MMP7	TGG-TGA-GTG-GTT-GGA-AAA-GC	AAC-ATT-CCC-TGA-TGC-CCT-TG
MXI1	CGA-TGG-AGA-AGC-ACA-TCA-AC	GTG-GGC-GAG-AAA-GAA-AGA-AC
	CCG-GGA-TGT-TTA-ATG-TGT-CC	AGG-TTG-CAA-GAT-CTG-ACA-CG
PPIG	GCC-TTC-CTT-TCT-GAT-AAC-CTA-GC	TGC-TTT-GTG-GAT-GCG-TTG-AC
PSA	GTT-GGG-AGT-GCA-AGG-AAA-AG	TAC-AAA-GCC-TCA-CGT-GCC-TA
	GCC-TTT-GTC-CCC-TAG-ATG-AA	CCA-GGA-GCC-CTA-TAA-AAC-CTT-C
PRY	ACA-AAT-CAC-AGT-GGG-GCA-AG	AGC-CAG-CCT-CAA-GAA-ACT-TC
RAD52	ATC-AAG-TCG-GAG-GGG-AAA-AG	AGC-GGC-TCA-GAA-CGT-AAA-AG
RBMY1B	CAG-ATG-AAT-TCC-ACC-TCA-GC	GAA-TAT-TGC-GGA-TCC-AGA-GG
	ATG-CCC-ACA-ATT-GCA-AAG-GG	ATC-ATC-TCG-CGG-TAA-CTC-TTC-C
RUNX2	GTG-GTG-GGT-GGG-ATT-ATG-TTT-G	CAC-CCA-CCC-TAT-TTC-TCT-TCA-G
	ATG-AGT-GGA-CGT-GTA-AGG-TAG-G	TTT-TCG-GTC-ACA-GGG-GAG-AG
SIN3A	GGA-ATC-CCC-CTT-TTC-TTT-CC	GTC-GGT-TTC-GGT-AGG-TTT-TG
	GCC-AAG-AGC-TCT-CCT-TCC-TG	CCA-ATC-TCA-GGC-GTT-TGG-G
TBCC	AAG-AAC-TGT-CCA-GCC-AAT-GC	AGG-GGC-AGT-TTC-AAA-GAC-TG
TOR3A	TTG-GTG-AGC-TGC-TGA-GAA-TG	AGC-ACC-GTG-TCT-CAC-AAA-TG

### RNA isolation and RT-qPCR

LNCaP and PC3 cells were transfected with 20pmol of control or ErbB3-specific siRNAs (Eurogentec) before total RNA was extracted using RNAble (Eurobio). cDNA were synthetized from 2–6μg of total RNA and amplified by qPCR as described above. GAPDH was used as internal control to normalize gene expression. List of cDNA RT-qPCR primers is given in Table 2.

**Table 2:** Primers for RT-qPCR analysis.

Gene Promoter Name	Forward primers (5'-3')	Reverse primers (5'-3')
ACE	CAT-CAC-CAC-AGA-GAC-CAG-CAA-G	CCG-CTT-GAT-AGT-GGT-GTT-CTG-C
ARID1A	AAG-CCA-CCA-ACT-CCA-GCA-TCC-A	CGC-TTC-TGG-AAT-GTG-GAG-TCA-C
CCND1	TCT-ACA-CCG-ACA-ACT-CCA-TCC-G	TCT-GGC-ATT-TTG-GAG-AGG-AAG-TG
CXCR3	ACG-AGA-GTG-ACT-CGT-GCT-GTA-C	GCA-GAA-AGA-GGA-GGC-TGT-AGA-G
ErbB2	GGA-AGT-ACA-CGA-TGC-GGA-GAC-T	ACC-TTC-CTC-AGC-TCC-GTC-TCT-T
GATA2	CAG-CAA-GGC-TCG-TTC-CTG-TTC-A	ATG-AGT-GGT-CGG-TTC-TGC-CCA-T
ID3	CAG-CTT-AGC-CAG-GTG-GAA-ATC-C	GTC-GTT-GGA-GAT-GAC-AAG-TTC-CG
MAP3K14	GGA-ATA-CCT-CCA-CTC-ACG-AAG-G	CTG-TGA-GCA-AGG-ACT-TTC-CCA-G
MXI1	AAA-GTG-GCG-ACT-GGA-ACA-GCT-G	GAA-CTC-TGT-GCT-TTC-AAC-ATC-CAC
PPIG	CTT-CAT-ACC-AGA-GAC-GAC-TTT-TAG	GCA-TCT-CTT-GCC-TCC-AAT-GTG-G
PRY	TGA-GAC-TAC-GGG-GAG-CAT-GTG-T	GTG-GAA-GTG-AGA-TAC-AGC-CAA-CC
RAD52	GCC-CAG-AAT-ACA-TAA-GTA-GCC-GC	CCA-CAT-TCT-GCT-GCG-TGA-TGG-A
RUNX2	CCC-AGT-ATG-AGA-GTA-GGT-GTC-C	CCC-TAA-GAC-TGG-TCA-TAG-GAC-C
SIN3A	CAG-AAT-GAC-ACC-AAG-GTC-CTG-AG	CAT-ACG-CAA-GTG-AGA-GGT-GTG-G
TBCC	GGC-TTC-AGA-GAC-GCG-AAC-AAG-A	CGA-GCA-AAG-GTG-GCG-ACG-AAA-A

## Results

### ErbB3 expression and localization in normal and tumour prostate tissues

**Fig 1:**
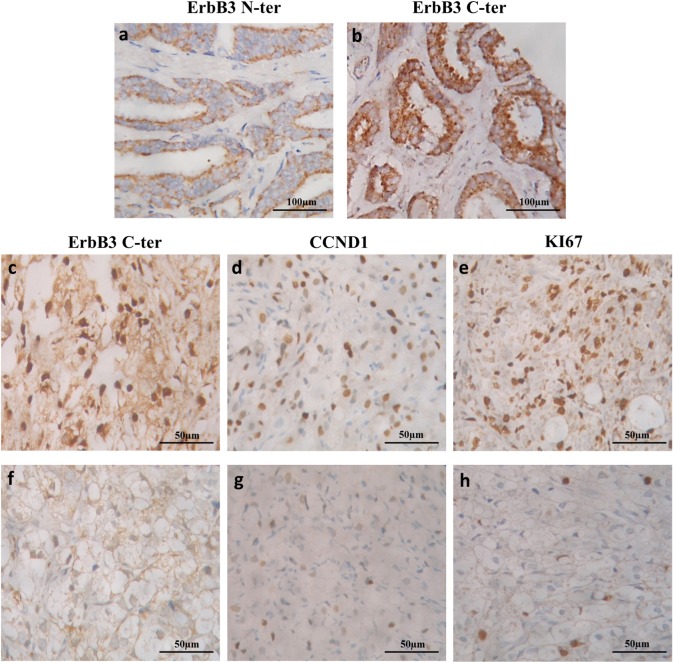
ErbB3 expression and localization in normal and tumour prostate tissues. Staining with ErbB3 N-ter antibody in PCa tissues was located at the membrane, without nuclear expression (a). No ErbB3 C-ter nuclear staining was detected in “normal” prostate tissues (b). Nuclear ErbB3 expression was observed in PCa cells, and more often in CRPC advanced tumours when compared to clinically localized cancers. In CRPC, high nuclear staining was associated with high CCND1 expression and tended to be associated with higher proliferation (Ki67) (CRPC 1, c-e), whereas most tumours without nuclear staining displayed low CCND1 expression and tended to show lower proliferation (CRPC 2, f-h).

137 HS, 32 CRPC and 50 normally appearing tumour-adjacent tissues were analysed by immunohistochemistry (Fig 1). In all samples, staining for ErbB3 N-ter, when present, was located mainly at the membrane, and no nuclear staining was observed (Fig 1A). All the 169 samples displayed cytoplasmic staining with ErbB3 C-ter. (Fig 1B). In contrast, nuclear staining was observed with ErbB3 C-ter in 60 of the 169 (35%) PCa samples, but in none of the non-tumour samples. Nuclear ErbB3 expression was most often observed in CRPC (17/ 32, 53%) than in HS tumours (43/ 137, 31%) (p = 0.03, Chi 2 test). In addition, when present, the percentage of PCa expressing ErbB3 in the nucleus was higher in CRPC (median 10%, range 2–95%) than in HS cancers (median 2%, range 1–20%) (p = 0.007, Mann Whitney test). The median expression of CCND1 was 15% of HS prostate cancer cells (range 0–70%, not shown) and 20% of cancer cells in CRPC tissues (range 0–80%) (Fig 1D and 1G). The median proliferation rate assessed by Ki67 staining was 1% of PCa cells in HS tissues (range 0–10%, not shown) and 17.5% in CRPC samples (Fig 1E and 1H). In HS, no significant association was observed between nuclear ErbB3, CCND1 (p = 0.09) and proliferation rate (p = 0.1, Spearman test). In CRPC, the nuclear expression of ErbB3 was significantly associated with *CCND1* expression, with a positive correlation (p = 0.01, Spearman test) (Fig 1C and 1D vs 1F and 1G). To resume, nuclear ErbB3 is highly and frequently expressed in CRPC and correlates with CCND1 expression, whereas no significant correlation was observed in HS tumours.

### ErbB3 expression and localization in prostate cell lines

**Fig 2:**
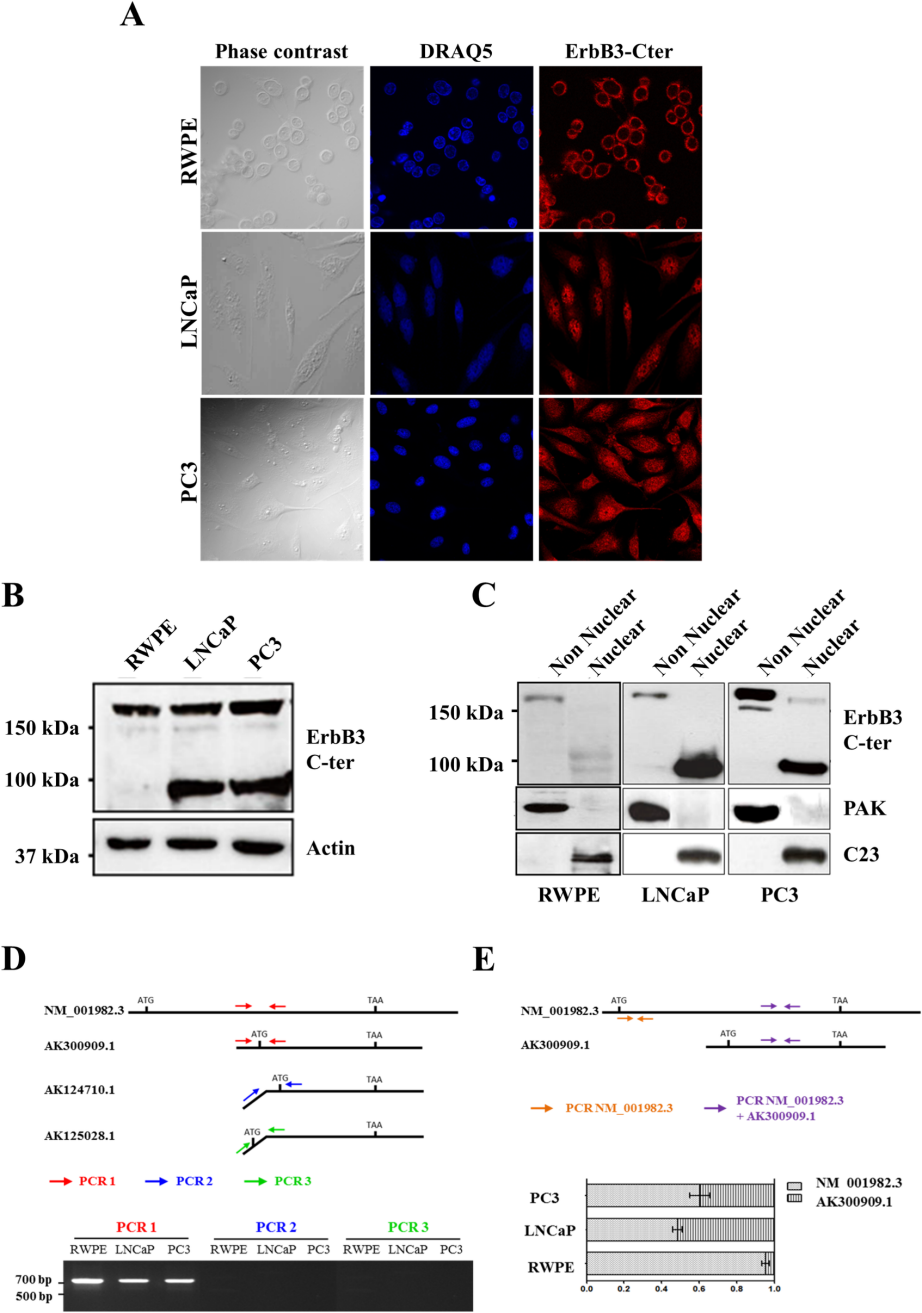
Characterization of an endogenous nuclear ErbB3 variant in prostate cell lines. (A) Both RWPE and PC3 cells display cytoplasmic and membrane ErbB3 localization. In addition, native PC3 cells exhibit a strong nuclear localization detected by the ErbB3 C-ter antibody (sc-285, Santa-cruz Biotechnology). (B) In total protein extracts, the ErbB3 C-ter antibody detects the full length ErbB3_185kDa_ protein in all cell lines and the 80kDa protein in the LNCaP and PC3 tumour cell lines. (C) The full length ErbB3_185kDa_ protein is almost only detected in the non-nuclear fractions that include plasma membrane and cytoplasmic proteins whereas a strong 80kDa signal is observed in the nuclear fractions of the tumour lines. (D) A 719 bp amplification product is detected in the three cell lines with the primers designed to amplify both the full length mRNA (NM_001982.3) and the AK300909.1 variant (PCR1), whereas PCR2 and PCR3 primers specific to AK124710.1 and AK1250281 respectively, failed to amplify any sequence. (E) Quantitative analysis with specific primers for ErbB3 transcripts indicate that NM_001982.3 and AK300909.1 are expressed at comparable levels in LNCaP and PC3 cell lines whereas AK300909.1 is slightly detectable in RWPE cells.

We next assessed endogenous ErbB3 expression and localization in prostate cell lines using ErbB3 C-ter antibodies. By indirect immunofluorescence, ErbB3 staining was mainly present at the plasma membrane and in the cytoplasm of RWPE cells whereas a high nuclear staining was detected in tumour cell lines (Fig 2A). Western blot analysis of total protein extracts showed that the three cell lines expressed similar levels of the full-length ErbB3_185kDa_ protein. An additional 80kDa protein was specifically detected in the hormone-sensitive (LNCaP) and hormone-resistant (PC3) tumour cell lines (Fig 2B). Next, western blotting was performed on non-nuclear vs nuclear protein fractions. The quality of the extracts was validated by the specific detection of the cytosolic PAK1 protein in the non-nuclear extracts and the C23 (nucleolin) protein in the nuclear extracts. As shown, ErbB3_185kDa_ was mainly expressed in the non-nuclear fractions and was absent or slightly detectable in the nuclear fractions of all cell types. Instead, the 80kDa protein was almost exclusively detected in the nuclear fractions of LNCaP and PC3 cells (Fig 2C). These data suggest that the nuclear protein detected in PCa cells could mainly correspond to the ErbB3_80kDa_ nuclear isoform previously described in lung cancer cell lines [17].

Among the putative variants reported in public databases that could be transcribed from the *ERBB3* gene, only three could correspond to the nuclear ErbB3_80kDa_ protein. They lack the 5’ sequence encoding the ligand-binding and transmembrane domains of the receptor and possess predicted nuclear localisation signals (NLS) [29]. To discriminate between those variants, we conducted RT-PCR with primers around the ATG starting codon of the mRNAs. In the three cell lines, a 719 bp PCR product was detected with the primers pair (PCR1) enabling the amplification of both the full length NM_001982.3 and the variant AK300909.1 transcripts that share the same nucleotide sequence. This experiment led us to eliminate AK124710.1 and AK1250281 and to focus on the AK300909.1 transcript (Fig 2D). This variant is predicted to encode a 699aa protein, whose sequence is strictly identical to the intracellular domain of ErbB3_185kDa_. To discriminate between NM_001982.3 and AK300909.1 expression in the cell lines, RT-qPCR was conducted using primers that amplify both transcripts or primers specific to the 5’ coding sequence of NM_001982.3 (Fig 2E). As shown, both transcripts were expressed at the same level in LNCaP and PC3 cells, whereas AK300909.1 was hardly detectable in RWPE cells (Fig 2E).

Thus, LNCaP and PC3 prostate tumour cell lines encode two different protein isoforms ErbB3_185kDa_ and ErbB3_80kDa_, corresponding to the NM_001982.3 and AK300909.1 mRNA transcripts respectively.

### Genome-wide analysis of ErbB3-target promoters in prostate cancer cells

**Fig 3:**
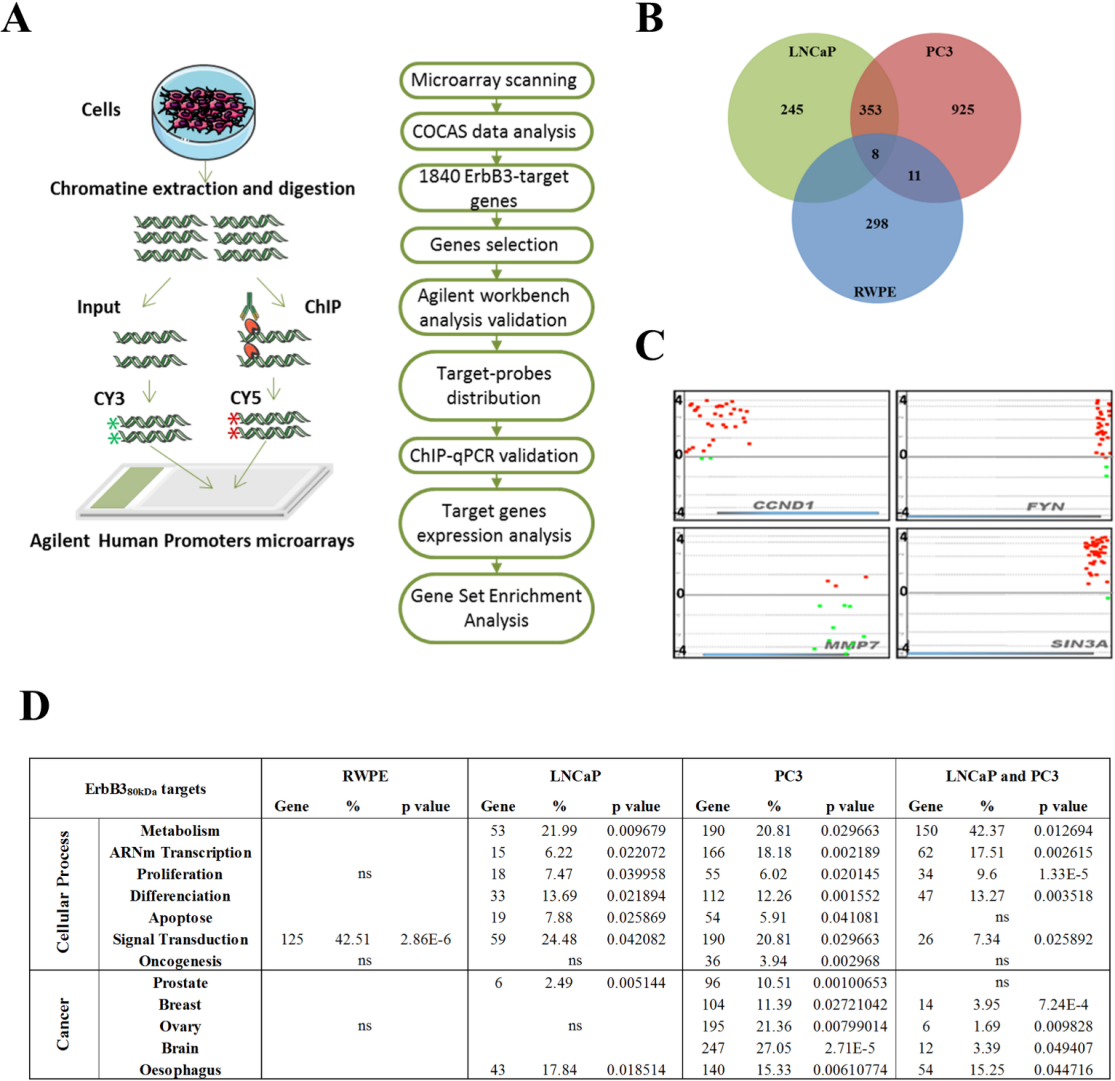
ChIP-on-chip workflow and Venn diagram. (A) Chromatin immunoprecipitation was performed upon endogenous conditions. Nuclear lysates were prepared from native cells grown in complete medium and without any androgen. ChIP-DNA and total DNA (Input) labelled with Cy5 and Cy3 fluorophores respectively, were co-hybridized on Agilent promoters tilling arrays. (B) Data analysis with CoCAS software led to select 1840 ErbB3-target promoters in the three cell lines. (C) Agilent Worbench 7.0 software analysis illustrating the distribution of positive probes (ErbB3-bound DNA/red dots) and negative probes (Input/ green dots) throughout the genes sequence (X-axis). Signal intensity is shown on the Y-axis. *CCND1* and *MMP7* correspond to positive and negative controls, respectively. (D) Gene ontology analysis of ErbB3-targets with DAVID.

To identify ErbB3-target genes, we next performed ChIP-on-chip assays using ChIP-validated ErbB3 C-ter antibodies [17] according to the workflow described (Fig 3A). ErbB3-bound genomic regions were first investigated using the standalone CoCAS V2.4 software [28]. After pre-processing step, peak detection algorithm detected high probe signals corresponding to significant changes in Cy3/Cy5 ratio of enriched genomic regions. A total of 68% of ErbB3-target probes were located into the core promoters, while 30% were inside the genes and 2% downstream of the transcription start site (TSS). Significantly enriched probes were identified above a threshold adjusted to 0.998, leading to finally identify 1840 ErbB3-target promoters distributed as follows: 317 promoters in RWPE, 606 in LNCaP and 1297 in PC3 cells, among which 8 were found in the 3 cell lines and 361 were shared by the LNCaP and PC3 cell lines (Fig 3B). The data were further confirmed with the Agilent Workbench 7.0 software. The distribution of the signal along the promoters was used to design ChIP-qPCR primers in the regions that displayed the highest concentration of ErbB3-enriched probes with the highest log-ratio (Fig 3C). The number of target promoters was significantly higher in hormone-resistant PC3 cells than in hormone-sensitive LNCaP cells, suggesting the involvement of ErbB3_80kDa_ in PCa progression and aggressiveness, in accordance with the data obtained in patients (Fig 1). Gene lists derived from ChIP-on-chip experiments were further analysed with DAVID (Database for Annotation, Visualization and Integrated Discovery) [30]. Functional annotation confirmed the involvement of ErbB3_80kDa_ targets in cellular processes highly deregulated in tumour mechanisms (Fig 3D). A significant number of ErbB3_80kDa_ targets in PC3 or PC3 and LNCaP tumour cell lines were already known to be associated with PCa, or other solid tumours, suggesting that ErbB3_80kDa_ could regulate in a more general way the mechanisms of tumour progression (Fig 3D).

### ChIP-qPCR validation of nuclear ErbB3 target promoters in prostate cancer cells

**Fig 4:**
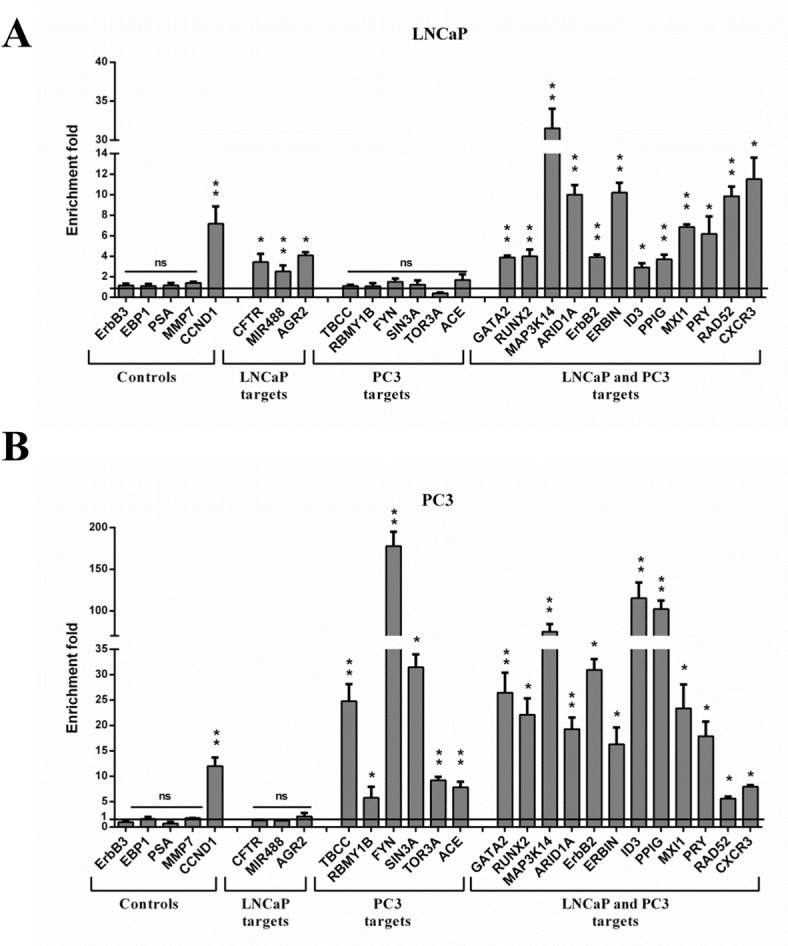
ChIP-qPCR validation. On the basis of biocomputing data, 16 randomly selected ErbB3-target promoters from LNCaP cells (A) and 19 randomly selected ErbB3-target promoters from PC3 cells (B) were tested. All showed a significant ErbB3 enrichment compared to the negative control ChIP-IgG (value normalized to 1). The *CCND1* promoter was used as a positive control whereas non-enriched promoters *ERBB3*, *EBP1*, *PSA*, and *MMP7* were used as negative controls. The data are representative of 3 independent experiments and are the means of triplicate samples. *P<0.05, **P<0.01, ns = non-significant, Student’s *t* test.

Among the 1840 targets, 26 genes were selected for ChIP-qPCR validation (Fig 4). Four non-enriched promoters (*ERBB3*, *EBP1*, *PSA* and *MMP7*) were used as negative controls and displayed no significant amplification, whereas the *CCND1* promoter displayed 7 to 12 enrichment fold in LNCaP and PC3 cells, respectively (Fig 4). The 3 LNCaP-specific targets exhibited significant enrichment fold in LNCaP cells and not in PC3 cells, whereas a strong and specific enrichment was observed for the 6 PC3-specific targets in the PC3 cell line (Fig 4A vs 4B). Enrichment fold observed for the shared promoters varied depending on the target and the cell line but was always higher in hormone-resistant PC3 cells than in LNCaP cells.

### Transcriptional regulation of ErbB3-dependent target genes

**Fig 5:**
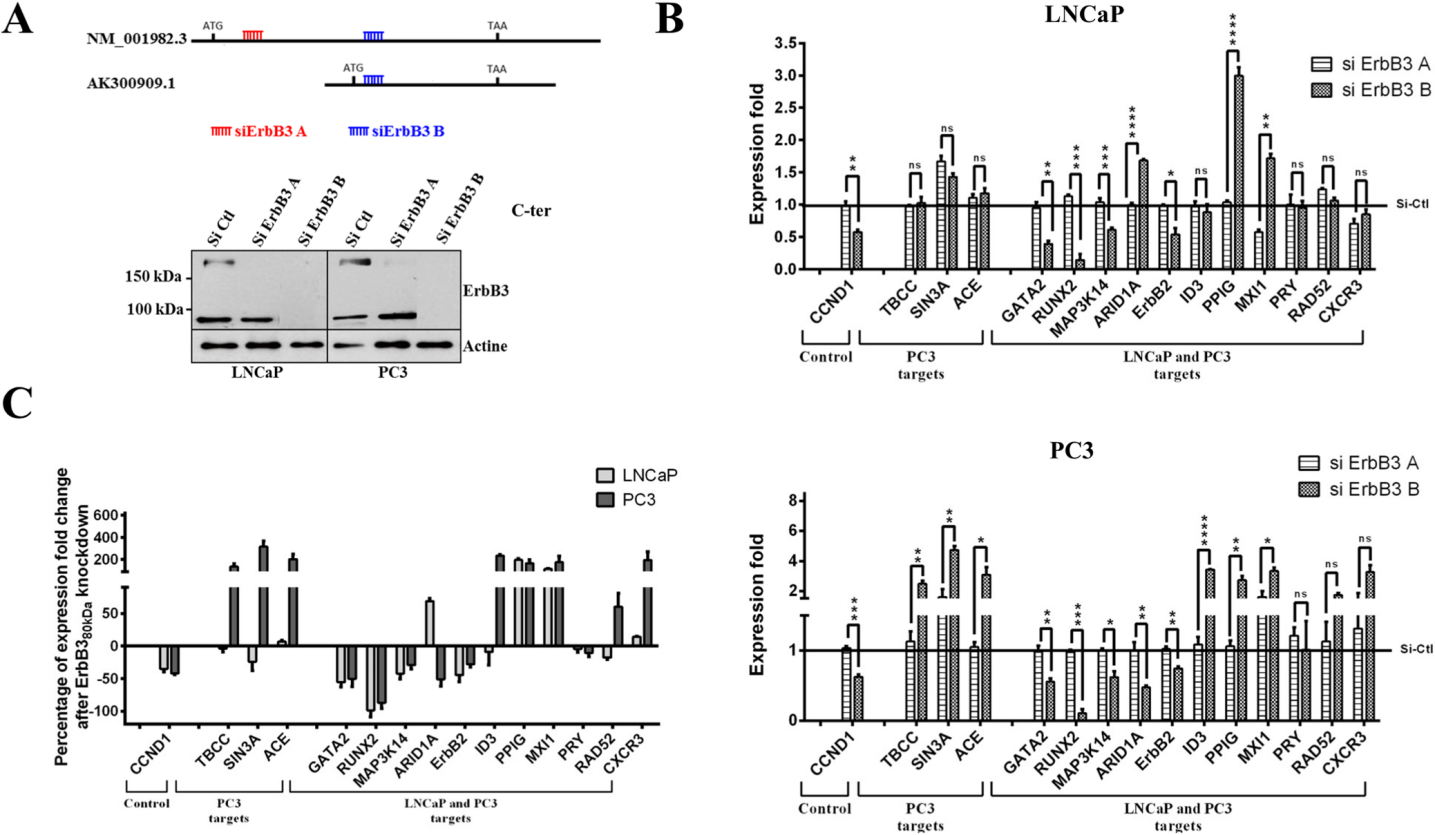
Gene expression profiling. (A) Two siRNAs were used to specifically inhibit the NM_001982.3 transcript (siErbB3A) or both the NM_001982.3 and AK300909.1 transcripts (siErbB3B) and validated by western blotting using the ErbB3 C-ter antibody. (B) LNCaP and PC3 cells were transiently transfected with siErbB3A or siErbB3B before mRNAs were reverse transcribed and amplified. RT-qPCR was performed on a selection of ErbB3-target genes. Expression fold change was normalised to control siRNA transfected samples. Transfections were done in triplicate, and the RT-qPCR results reported are from at least three independent experiments. (C) Expression fold change consecutive to the inhibition of the ErbB3_80kDa_ isoform in each cell line and for each target gene is calculated by the ratio (relative gene expression upon siErbB3B treatment)/(relative gene expression upon siErbB3A treatment). ErbB3_80kDa_-dependent transcriptional activation of *GATA2*, *RUNX2*, *MAP3K14*, *ErbB2* and *CCND1* and inactivation of *PPIG*, MXI1 were similar in LNCaP and PC3 whereas *ARID1A*, *TBCC*, *FYN*, *SIN3A*, *TOR3A*, *ACE*, *RAD52*, *CXCR3A* appeared to be differentially regulated in the two cell lines. *P<0.05, **P<0.01, ns = non-significant, Student’s *t* test.

Given the structure of the AK300909.1 transcript, no siRNAs specific to this variant can be designed. So, we addressed the transcriptional effects of ErbB3_80kDa_ by subtractive analysis: two types of siRNAs were used, targeting either the 5’ coding sequence of the NM_001982.3 *ERBB3* transcript (siErbB3A) or the 3’ coding sequence of both NM_001982.3 and AK300909.1 *ERBB3* transcripts (siErbB3B) as indicated on Fig 5A. The siRNAs were validated by RT-qPCR (not shown) and western blotting analysis: ErbB3_185kDa_ expression was inhibited by both siRNAs, whereas ErbB3_80kDa_ expression was only decreased by siErbB3B in LNCaP and PC3 cell lines (Fig 5A). Under these conditions, we next analysed the expression of 15 ErbB3_80kDa_-targets genes (Fig 5B). As expected, the expression of *CCND1* was significantly reduced in LNCaP and PC3 cells transfected with siErbB3B vs siErbB3A, the latter displayed no difference with the control siRNA. The same applied for *GATA2*, *RUNX2*, *MAP3K14* and *ErbB2* in the two cell lines, and for *ARID1A* in PC3 cells. Conversely, siErbB3B induced higher expression for *PPIG* and *MXI1* in the two cell lines and for *ID3* and PC3-only targets in PC3 cells (Fig 5B). To simplify data analysis, the percentage of expression fold change that can be specifically attributed to the nuclear isoform was calculated with the formula (relative gene expression upon siErbB3B treatment)/(relative gene expression upon siErbB3A treatment) for each target gene (Fig 5C). As shown, ErbB3_80kDa_ transcriptionally activates *CCND1*, *GATA2*, *RUNX2*, *MAP3K14* and inhibits *PPIG*, *MXI1* in both LNCaP and PC3 cells, whereas it inhibits *TBCC*, *SIN3A*, *ACE*, *ID3*, *RAD52*, *CXCR3* in PC3 cells only. Western blot analysis performed on ErbB3_80kDa_-targets confirmed the transcriptional effects of the nuclear ErbB3 isoform at the protein level (S1 Fig).

**Fig 6:**
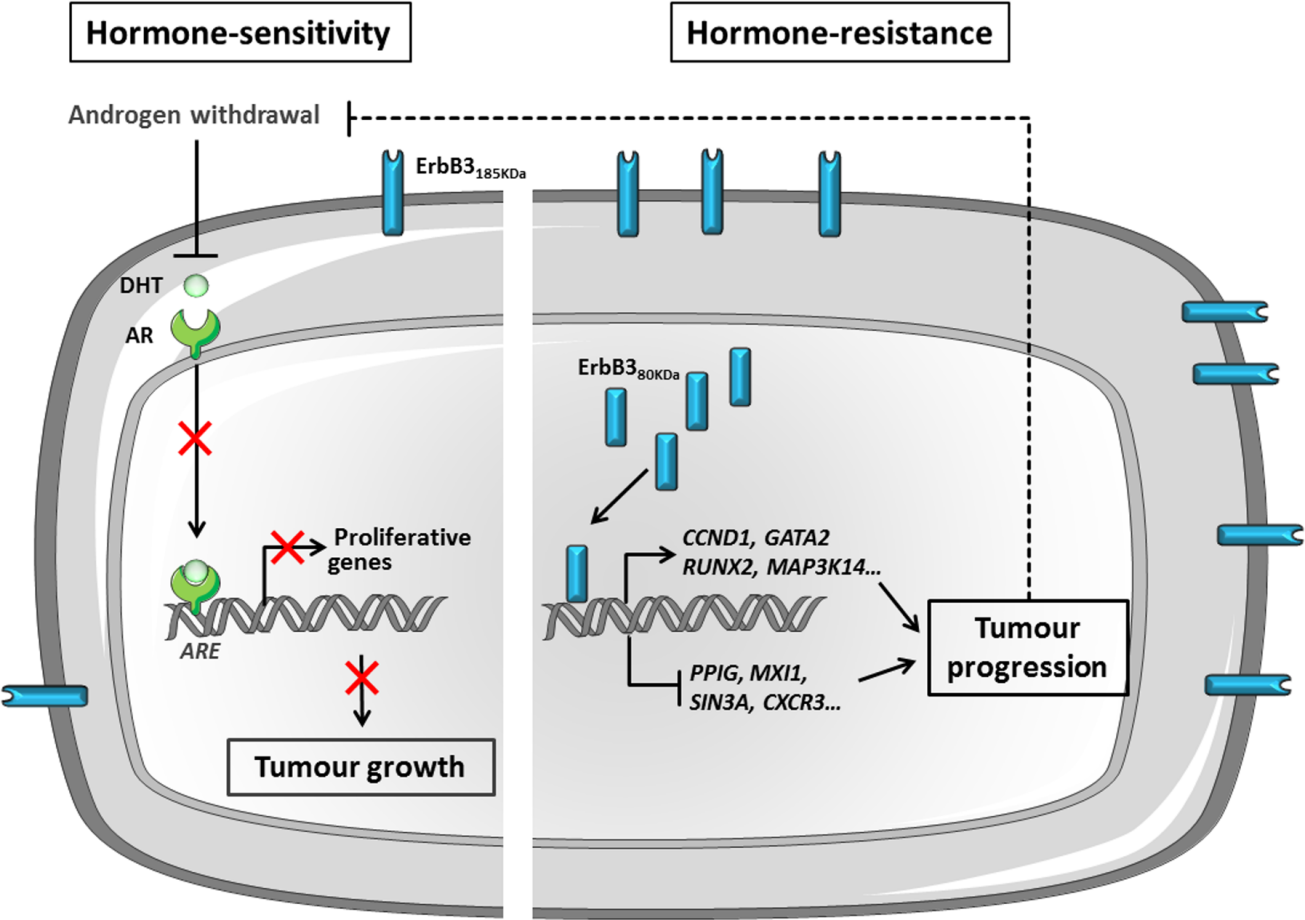
Proposed model for ErbB3_80kDa_ functions in prostate cancer progression. Blocking RA-dependent signalling by androgen withdrawal therapy (AWT) first inhibits tumour growth, but soon alternative proliferative pathways are reactivated inducing tumour progression. Increased ErbB3 expression and subsequent reactivation of the PI3K pathway in response to AWT is a major mechanism of tumour resistance. Alternatively, we report here the nuclear accumulation of ErbB3_80kDa,_ a variant lacking the ligand-binding and transmembrane domain of ErbB3_180kDa_, in advanced PCa resistant to therapy (CRPC). As a co-regulator for the transcription of genes associated with cell proliferation, differentiation, apoptosis, oncogenesis, ErbB3_80kDa_ is likely to be strongly involved in therapy resistance and progression to fatal PCa. *DHT*: *dihydrotestosterone; AR*: *androgen receptor; ARE*: *androgen responsive elements*.

The data presented here can be integrated to propose a model for nuclear ErbB3_80kDa_ functions in prostate cancer progression (Fig 6).

## Discussion

The goal of this study was to bring out molecular pathways involving ErbB3 in tumour progression and metastatic spreading, in order to characterize new potential therapeutic targets for prostate cancer. Recent therapies approved for recurrent prostate cancer delay metastatic progression and extend overall survival, but still fail to inhibit tumour progression to fatal CRPC. Inappropriate expression and/or activation of the ErbB family of tyrosine kinase receptors has been reported in advanced PCa, leading to consider ErbB1, ErbB2 and ErbB3 as potential diagnostic and prognostic markers [22,31]. Accordingly, increased ErbB3 expression has been reported in androgen-dependent PCa treated by androgen withdrawal and was associated with poor prognosis. This overexpression correlates with cell cycle progression and reactivation of the transcriptional activity of the AR in absence of androgens [26]. In addition, the nuclear compartmentalization of ErbB3 has been suggested to play a role in PCa progression but the mechanisms involved still remain unknown [18,27,32].

We first investigated the expression of ErbB3 on normal or tumour prostate tissues. Staining for ErbB3 N-ter was directed at the membrane, without nuclear expression. Using the ErbB3 C-ter antibody, the nuclear staining we observed was restricted to PCa cells and was increased in advanced castration-resistant prostate cancer when compared to localized tumours. These data strengthen the involvement the nuclear ErbB3 protein in PCa progression up to terminal castration-resistant stage [18]. As we failed to detect nuclear staining in CRPC with anti ErbB3 N-ter antibodies, we assumed that the nuclear ErbB3 protein could be a variant, on the model of the previously described ErBB3_80kDa_ or ErbB3_50kDa_ isoforms [16, 17]. ErbB3 protein expression assessed on normal and tumour prostate cell lines confirmed our hypothesis, with the full length receptor ErbB3_185kDa_ mainly expressed in the non-nuclear protein fraction of all cell lines and an ErbB3_80kDa_ isoform mainly detected in the nuclear protein fraction of the tumour cell lines. To date, only the full length ErbB3_185kDa_ protein was reported in the nucleus of prostate tumours and cell lines [15,19]. The mechanisms of nuclear localization analysed so far involved routing from endosomes and/or membrane cleavage following receptor activation [5–7, 32–36]. Since ErbB3_185kDa_ is tyrosine kinase deficient, its phosphorylation occurs upon dimerization with other members of the ErbB family [37]. However, heregulin1ß treatment of PCa cell lines led to phosphorylate cytoplasmic but not nuclear ErbB3 protein [18]_._ Furthermore, nuclear translocation of ErbB1 or ErbB2 together with ErbB3, has never been reported in the nucleus of PCa cells, suggesting that the mechanisms underlying the nuclear localization of the full length ErbB3_185kDa_ receptor may differ from those already described [5,6]. Alternatively, the nuclear ErbB3 protein detected in PCa cells would rather correspond to ErbB3_80kDa,_ on the same model as ErbB3_55kDa_ was specifically detected in the nucleus of normal Schwann cells [16].

ChIP-on-chip experiments performed to address the function of ErbB3_80kDa_ in PCa progression revealed that the number of ErbB3_80kDa_ target promoters was significantly higher in aggressive PC3 cells than in LNCaP hormone-sensitive cells, matching with the increased ErbB3 nuclear staining observed in castration resistant prostate tumours (Fig 1 and [18]). Genes set enrichment analysis also indicated that ErbB3_80kDa_ target genes were involved in a broad range of cellular processes such as transcription, proliferation, differentiation, apoptosis, and most of these genes are deregulated in neoplastic tissues. Interestingly, PC3-specific target genes were directly related to oncogenic processes in functional annotate on analysis, suggesting that these targets could be leading players in progression up to fatal PCa. Bioinformatics data were strengthened by the validation of 26 selected genes as effective ErbB3_80kDa_-bound targets. To do so, we had to deal with the fact that *CCND1* in lung and breast tumour cell lines, *EZR* (Ezrin) and *HMGB1* in normal Schwann cells, were the only published nuclear targets of ErbB3 [16,17,19]. The *CCND1* promoter we had previously identified as a transcriptional target of ErbB3_80kDa_ in the H358 lung carcinoma cell line, was found significantly enriched in LNCaP and PC3 cell lines and was considered further as positive control for ErbB3_80kDa_ binding [17]. On this basis, ChIP-qPCR analysis first indicated that the enrichment fold was promoter- and cell line- dependent. Indeed, no enrichment was found for the PC3-specific targets *TBCC*, *FYN* or *ACE* in LNCaP cells (Fig 4A), therefore no variation was observed at the expression level (Fig 5B). More, the amount of ErbB3_80kDa_ linked to the promoters was significantly higher in PC3 cells than in LNCaP. The latter point might be explained by the fact that ErbB3_80kDa_ doesn’t present any feature of a transcription factor. Thus, ErbB3_80kDa_ is more likely to bind the target promoters in an indirect manner. The recruiting factors may be different or differentially expressed between the LNCaP and PC3 cells. This could explain the discrepancies observed in the amount of ErbB3_80kDa_ on target promoters and also in the number of targets. Characterization of ErbB3_80kDa_ partners involved in the complex could offer interesting insights for the development of new therapies.

Targets characterized in this study can be classified into two categories whether they are already known to be involved in PCa or not. In this way, *RUNX2*, *CXCR3* or *GATA2* seem to be major players of progression and metastatic dissemination in PCa [38–43] whereas *MXI1* has been recently identified as a new biomarker of aggressive disease [44]. In contrast, MAP3K14, also known as NF-ĸB-inducing kinase (or NIK) is a major player of the non-canonical NF-κB pathway mainly associated with lymphoid malignancies [45,46]. Interestingly, the non-canonical NF-κB involves specific membrane receptors among which the receptor activator for nuclear factor kB (RANK) [46].

Bone metastasis is the primary cause of mortality in patients with prostate cancer. Normal bone remodelling relies on a balance between bone resorption by osteoclasts and bone formation by osteoblasts. The RANK/RANKL pathway controls precursor’s differentiation into mature osteoclasts that promote bone resorption. Metastatic prostate tumour cells alter this balance by producing paracrine factors that stimulate neighbouring osteoclasts to resorb bone and in turn, bone resorption stimulates tumour cells growth [47]. Thus, increased MAP3K14 transcription by ErbB3_80kDa_ is strongly to be involved in bone resorption during prostate cancer progression, through constitutive activation of the non-canonical NF-ĸB signalling pathway.

These data strengthen the validity of our study and raise the question of the relevance of therapies in advanced PCa. At the time of personalized therapy, targeting ErbB3 could be attractive. Neutralizing antibodies specific for the extracellular domain of ErbB3 receptors are currently under evaluation in phase I or II clinical trials in various tumours except prostate. As prostate tumours develop resistance therapy through compensatory pathways regulating ErbB3 expression and localization, anti-ErbB3 therapeutic antibodies could be used for tumours displaying ErbB3_185kDa_ overexpression and/or nuclear localization but not for tumour expressing ErbB3_80kDa_ [17, 25]. Indeed, as devoid of extracellular ligand-binding domain, ErbB3_80kDa_ is untargeted by ErbB3 therapeutic antibodies currently under Phase I or II clinical assays in various tumours, we have tested so far (unpublished data). The potential use of siRNA targeting specifically the ErbB3_80kDa_ isoform is not possible here as the full-length RNA would also be downregulated. Therefore, patients with ErbB3_80kDa_ positive tumours should be proposed more appropriate treatments aiming at inactivate the downstream targets rather than the nuclear ErbB3_80kDa_ protein. In this context, recently described markers such as GATA2, a poor prognosis factor associated with early relapse, could be of interest [42]. Recently, nanoparticles carrying siRNA targeting GATA2 significantly reduced tumour growth in a xenograft murine model of NSCLC harbouring oncogenic KRAS mutations [48].These promising results require further exploration. Targeting the non-canonical NF-kB could also be explored. Inhibition of the RANK-RANKL axis in association with the bone morphogenetic protein (BMP) activity has yet been reported in a murine model of lytic prostate cancer lesion in bone. The combine treatment led to inhibit osteoclast-dependent bone resorption and to reduce bone lesion formation [49].

Therefore, multitargeted approaches aiming at inhibit several ErbB3_80kDa_-regulated factors according to the pattern of the tumour, could offer interesting individual perspectives for patients.

## Supporting Information

S1 Fig Transcriptional effects of the nuclear ErbB3_80kDa_ isoform at the protein level. PC3 cells were transiently transfected with si control, siErbB3A or siErbB3B 48h before cytosolic (C) and nuclear (N) extraction. Western blotting was performed using antibodies to PPIG (sc-100699, SCBT), GATA2 (sc-9008, SCBT), RUNX2 (sc-10758, SCBT), CCND1 (#2922, CST) or histone H3 (#2650, CST) as a control for nuclear extraction.
(PPTX)
