# Supplementary material for: Integrated and Functional Genomics Analysis Validates the Relevance of the Nuclear Variant ErbB380kDa in Prostate Cancer Progression
Source: PLoS One. 2016 May 18;11(5):e0155950. doi: 10.1371/journal.pone.0155950 (PMC4871423; doi:10.1371/journal.pone.0155950)
Supplement: S1 Fig — PC3 cells were transiently transfected with si control, siErbB3A or siErbB3B 48h before cytosolic (C) and nuclear (N) extraction. Western blotting was performed using antibodies to PPIG (sc-100699, SCBT), GATA2 (sc-9008, SCBT), RUNX2 (sc-10758, SCBT), CCND1 (#2922, CST) or histone H3 (#2650, CST) as a control for nuclear extraction. (PPTX) [file pone.0155950.s001.pptx]

## Slide 1
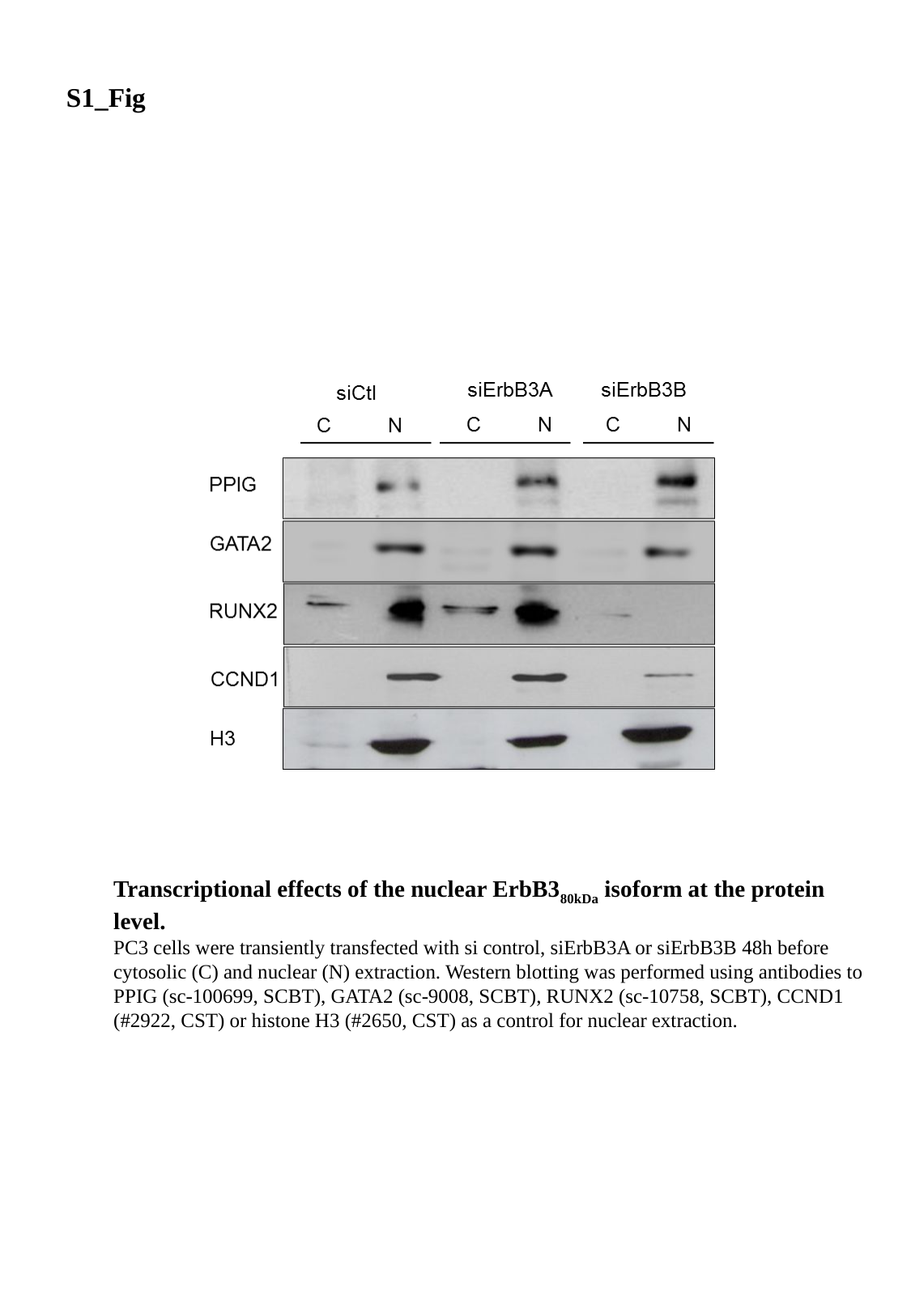

S1_Fig
Transcriptional effects of the nuclear ErbB380kDa isoform at the protein level.
PC3 cells were transiently transfected with si control, siErbB3A or siErbB3B 48h before cytosolic (C) and nuclear (N) extraction. Western blotting was performed using antibodies to PPIG (sc-100699, SCBT), GATA2 (sc-9008, SCBT), RUNX2 (sc-10758, SCBT), CCND1 (#2922, CST) or histone H3 (#2650, CST) as a control for nuclear extraction.
